# Correction to “Understanding the variability in red cell and plasma volume combinations can help guide management in heart failure”

**DOI:** 10.1002/ehf2.15291

**Published:** 2025-04-08

Miller WL , Fudim M , Kittipibul V , Yaranov DM , Carry BA , Silver MA . Understanding the variability in red cell and plasma volume combinations can help guide management in heart failure. ESC Heart Failure. 2025 Feb;12(1):142–9.39267242 10.1002/ehf2.15070PMC11769611

The second to the last author, Brendan A. Carry, is incorrectly spelled. The author's name correctly spelled is Brendan J. Carry.

We apologize for this error.

